# Practice patterns in the management of patients with differentiated thyroid cancer in Ontario Canada 2000-2008

**DOI:** 10.1186/s40463-014-0029-3

**Published:** 2014-07-24

**Authors:** Stephen F Hall, Jonathan C Irish, Patti A Groome, David R Urbach

**Affiliations:** 1 Departments of Otolaryngology and Oncology, Division of Cancer Care and Epidemiology, Queen’s Cancer Research Institute, Queen’s University, 10 Stuart St. 613 533 6000 ext 78535, Kingston K7L 3 N6, ON, Canada; 2 Department of Otolaryngology, Wharton Head & Neck Centre, University Health Network, Princess Margaret Hospital, 610 University Ave., Toronto M5G 2 M9, ON, Canada; 3 Division of Cancer Care and Epidemiology, Queen’s Cancer Research Institute, 10 Stuart St, Kingston, ON, Canada; 4 Division of General Surgery, Toronto General Hospital, University Health Network, 200 Elizabeth St, Toronto M5G 2C4, ON, Canada

**Keywords:** Thyroid cancer, Population-based study, Patterns of practice, Surgery, Radioactive iodine 131

## Abstract

**Background:**

The extent of treatment for differentiated thyroid cancer remains controversial. The objective of this study was to describe the variations in practice prior to diagnosis and for the first year after diagnosis, including the investigations, the extent of surgery and the use of RAI 131, for all patients with thyroid cancer (TC) treated Jan 1 2000 to Dec 2008 across Ontario Canada.

**Method:**

Population-based study of all patients who had a therapeutic surgical procedure for TC based on the data holdings of the Institute of Clinical Investigative Sciences (ICES) linking the Ontario Cancer Registry to the Ontario Health Insurance Plan and to the Canadian Institutes of Health Information. The analysis includes comparisons between health care utilization/geographic regions and between treating specialties. The study population was 12957 patients.

**Results:**

There was a 112% increase in case detection over 9 years. Overall the initial (index) surgery was less-than-total thyroidectomy (LTT) in 37.6% and 63.4% of the patients who had total thyroidectomy (TT) as an index surgery went on to adjuvant RAI, however there was wide variation in all aspects of patient care across the province, between Local Health Networks and between surgical specialties.

**Conclusion:**

In Ontario, there was wide variation for most aspects of the management of TC and, as the incidence of TC is increasing at least 7% per year in females, these data provide a foundation for future discussions, the provision of health care services and research.

## Introduction

When doctors are uncertain and there is a lack of consensus on best treatment, practice will vary [[1],[2]]. As there is no randomized control trial evidence for any aspect of the treatment of differentiated thyroid cancer, the treatment decisions on the extent of both surgical and use of post operative radio active iodine 131 (RAI) remain controversial [[3],[4]] and treatment is known to vary. In the absence of evidence, a first step towards discussion and consensus is to understand current practice and the objective of this project was to describe the management and the variation in the management of patients with thyroid cancer from Ontario Canada during their first year after diagnosis based on a complete unbiased patient population.

## Methods

### Study population and data sources

We identified all patients over 18 years of age with a diagnosis of thyroid cancer (ICD 193) in the Ontario Cancer Registry (OCR) between Jan 1 1999 and Dec 31 2008 who had a surgical procedure for thyroid cancer between Jan 1 2000 and Dec 31 2008 (n = 13724). The OCR is a population-based tumor registry operated by Cancer Care Ontario and consists of linked data on all patients with cancer including demographic information from all cancer treatment centers and all pathology reports of cancer from all hospitals and laboratories. This cohort was linked at the Institute of Clinical and Evaluative Sciences (ICES) to physician billing codes and dates of treatment or testing (surgery, diagnostic radiology, radioactive iodine, and fine needle aspirate biopsy (FNAB)) in the Ontario Health Insurance Plan (OHIP) administrative database and to hospital procedure codes and dates of treatments (surgery, in-patient radioactive iodine) in the Canadian Institutes of Health Information (CIHI) databases. We used the CIHI hospital procedure codes as our primary data source for extent of surgery. We compared the CIHI hospital procedure codes to the OHIP surgical billing codes, compared the sequences of codes, assigned an initial index surgery to each patient and excluded 769 cases due to discrepancy on the extent of the index surgery, incomplete information or incompatible codes. Patients with biopsies, nodulectomy or other non- therapeutic procedure were excluded. Hemithyroidectomy and subtotal thyroidectomy were combined as “less than total thyroidectomy” (LTT) to compare with total thyroidectomy (TT). The date of diagnosis for each patient was the date of the index surgery and the sequence of treatment for each patient included index surgery, completion surgery, subsequent surgery (after completion or total thyroidectomy), neck dissection (any neck dissection billing code) and first radioactive iodine ablation (RAI) within the first year after the index surgery. Histological subtypes for this study as assigned at the OCR included differentiated (papillary and follicular), medullary and other (insular and anaplastic). The study population was 12,959 patients treated with an index surgical procedure for thyroid cancer. The same dataset was used for a previous publication Access, Excess and OverDiagnosis: the case for thyroid cancer [[5]].

**Figure 1:**
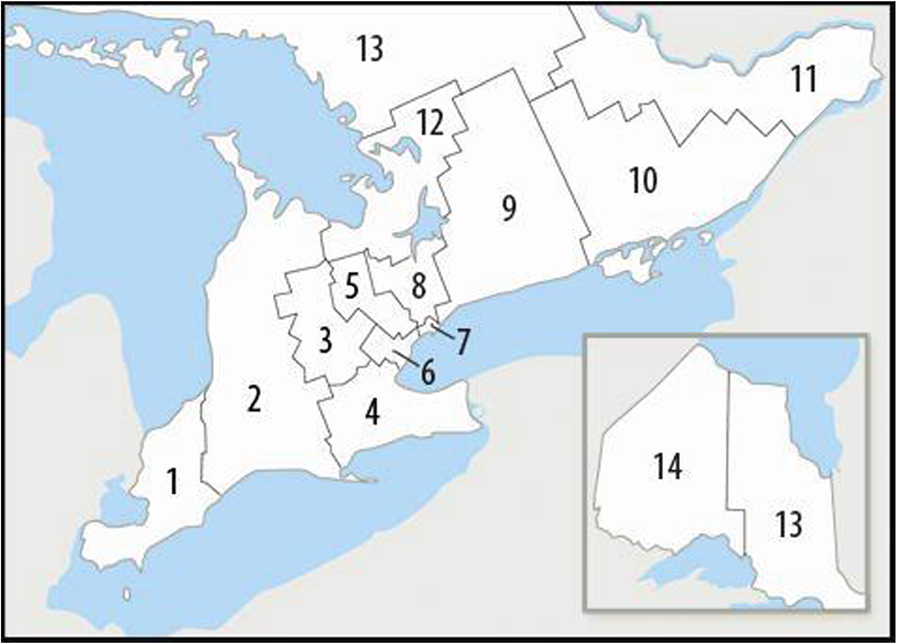
LHINs of Ontario.

Health care services in the Province of Ontario Canada are divided into 14 geographic regions (Local Health Integration Networks (LHINs)) (Figure 1) based on healthcare utilization as well as political boundaries. Toronto is the largest city in the Province of Ontario and the Greater Toronto Area (GTA) is within LHINs 5, 6, 7, 8 and 9. These LHINs also include communities outside and surrounding the GTA.

### Ethical approval

This study was approved by the ICES Cancer Program and the Queen’s University Research Ethics Board #OTOL-041-10.

### Analysis

We calculated the mean rates of diagnosis per 100,000 (the total number of cases for 8 years for the total population of those years) and compared them by LHIN. The 95% confidence intervals are based on the mean of the rates over the 9 years assuming a Poisson distribution. There were a small number of cases per year in some LHINs and because we used the rates based on the average over 9 years, age and gender adjustment was not practical. We examined the age and gender indirectly standardized incidence ratios for each LHIN compared to the population of all Ontario in 2004 and found that the adjusted ratios reflected the actual rates.

There is no restriction in Ontario on where patients can be treated. Referral patterns can be variable, resources vary and it is common practice for patients to receive treatment outside of their LHIN of residence. To understand the patterns of practice we therefore report the LHIN of the patient’s residence to compare rates of diagnosis and report the both LHIN of the treatment and LHIN of residence to describe and compare the variability of practice.

## Results

### Overall

Eighty percent of patients were female and the average age for females was 47.1, 3.5 years younger than males. Papillary and follicular carcinoma accounted for 98.0% of cases with a histological diagnosis code noting that the code in the OCR was missing for 6.3% of cases (n = 815). There were only 184 cases of Medullary carcinoma and 57 cases of Anaplastic/Insular carcinoma over the 9 years.

**Figure 2:**
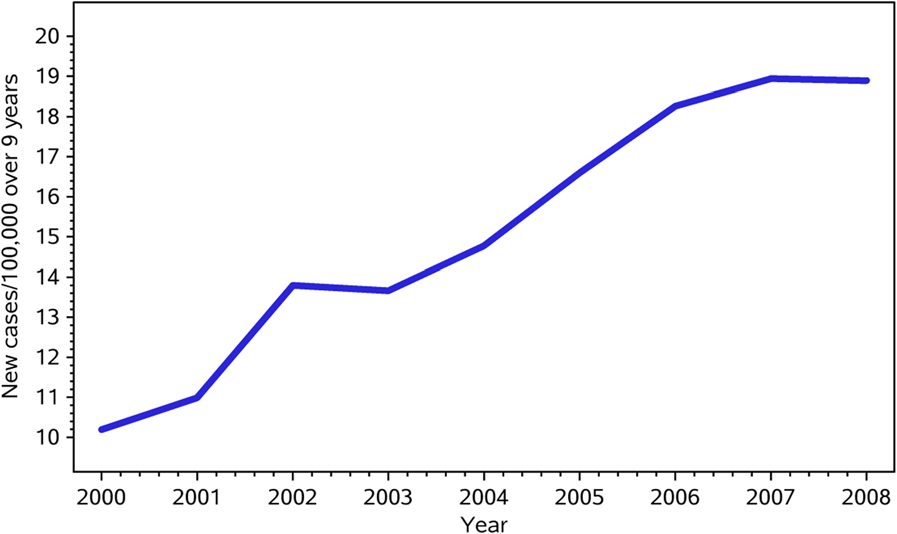
New cases per 100,000 by year in Ontario.

**Figure 3:**
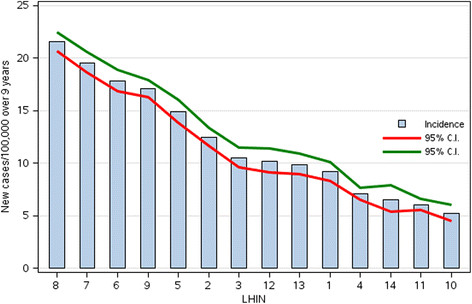
Diagnosis rate (per 100,000) over 9 years by LHINs.

The number of cases per year increased from 893 to 1890 per year creating an overall increase of 112% (Figure 2) due to a rate increase from 10.19/100,000 to 18.89/100,000 across Ontario over the 9 years. There was a wide variation across the Province in the rate of diagnosis of incident cases by LHIN (Figure 3) with some of the GTA LHINs having diagnosis rates up to 4 times greater than other regions of the province. With the indirect age/gender standardized adjustment the rank order of the LHINs on Figure 3 did not change.

### Investigations

#### FNAB

79% of patients had at least one FNAB prior to index surgery and this proportion stayed constant over the 9 years despite the doubling of the numbers of FNABs per year in Ontario with increasing incidence of thyroid cancer. Patients over 70 years of age had fewer FNAs and patients with differentiated thyroid cancer had more FNABs than the other histological types (p = 0.001).

#### Diagnostic ultrasound

**Figure 4:**
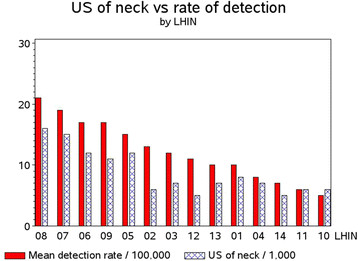
Rate of Neck Ultrasounds vs Detection of Thyroid Cancer by LHIN.

With the progressive rise in the rate of diagnosis of thyroid cancer, the rates of diagnostic ultrasound of the neck for the general population increased across the Province from 681/100,000 to 1477/100,000 over the 9 years. Figure 4 compares the rates of detection by LHIN to rates of diagnostic ultrasound of the neck by LHIN. For Figure 4, the thyroid cancer cohort was excluded and only one ultrasound per person was counted. The relationship is very clear with differences in physician behaviour for ordering ultrasound imaging in different LHINs creating differences in rates of diagnosis (r = 0.91).

### Index surgery

The index (initial) surgery was LTT for 37.6% (4864) and TT for 62.4% (8095) of cases. A neck dissection at the time of index surgery was recorded in 14.9% of cases (n = 1931). Almost all patients with medullary and anaplastic/insular carcinoma had total thyroidectomy.

**Figure 5:**
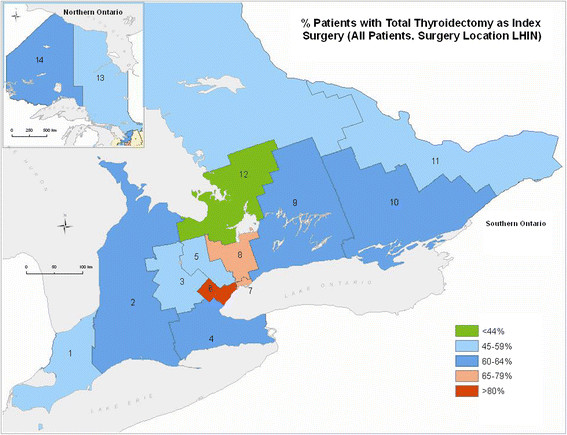
Extent of index thyroid surgery: % of patients with total thyroidectomy based on treatment LHIN.

There were large variations in the extent of index surgery across Ontario. Figure 5 presents the rates of TT when the index surgery (29% to 82%) performed in the various LHINs across Ontario. There was substantial across-LHIN patient migration for surgery (33% of all Ontario patients had surgery outside of their residence LHIN and 40.5% of patients living in a GTA LHIN had surgery in another GTA LHIN) resulting in 28% of LHIN 6 surgical cases migrating in from another LHIN yet only 28% of the thyroid cancer patients who lived in LHIN 8 had their surgery in LHIN 8.

### Surgeons

**Figure 6:**
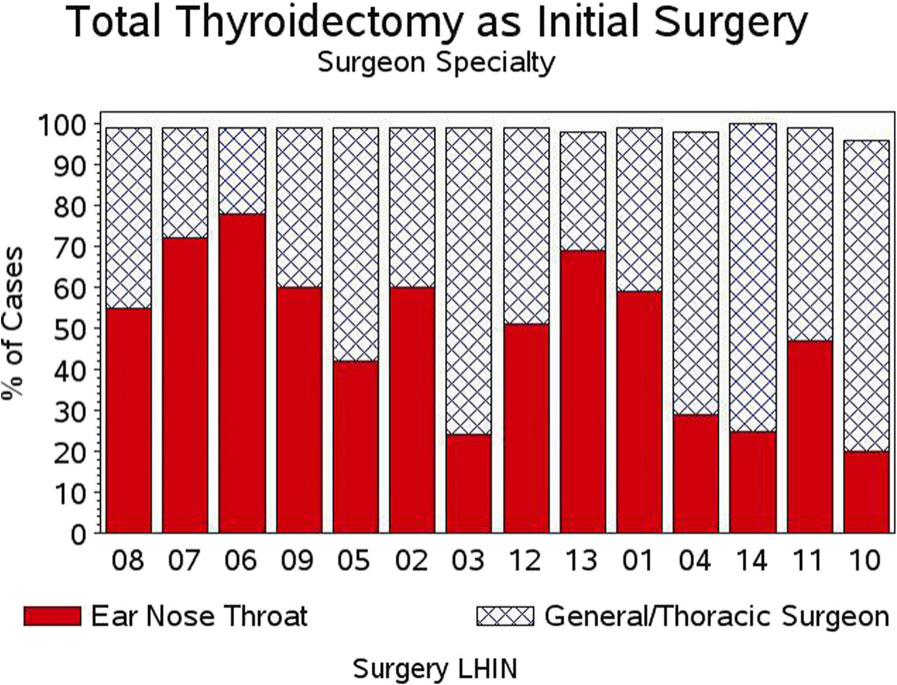
Extent of initial surgery: % of cases having total thyroidectomy by specialty by LHIN of treatment.

Overall 57.0% of the initial index surgical cases were done by Otolaryngologist/Head and Neck Surgeons and 42.7% by General Surgery/Thoracic Surgery (34 unknown). Figure 6 compares the extent of index surgery by both specialty and by LHIN, with the LHINs ranked in the order of thyroid cancer detection (Figure 3). There were large differences in the extent of index surgery between the surgical specialty groups with a pattern of TT being done more commonly by Otolaryngologists in regions of higher thyroid cancer detection.

### Subsequent surgery

54.2% of the 4864 patients who had a LTT procedure as index surgery went on to completion thyroidectomy whereas only 18 of the 8085 patients (0.002%) who had TT as index surgery had a subsequent surgery on thyroid or neck in the first year.

### Radioactive iodine ablation

**Figure 7:**
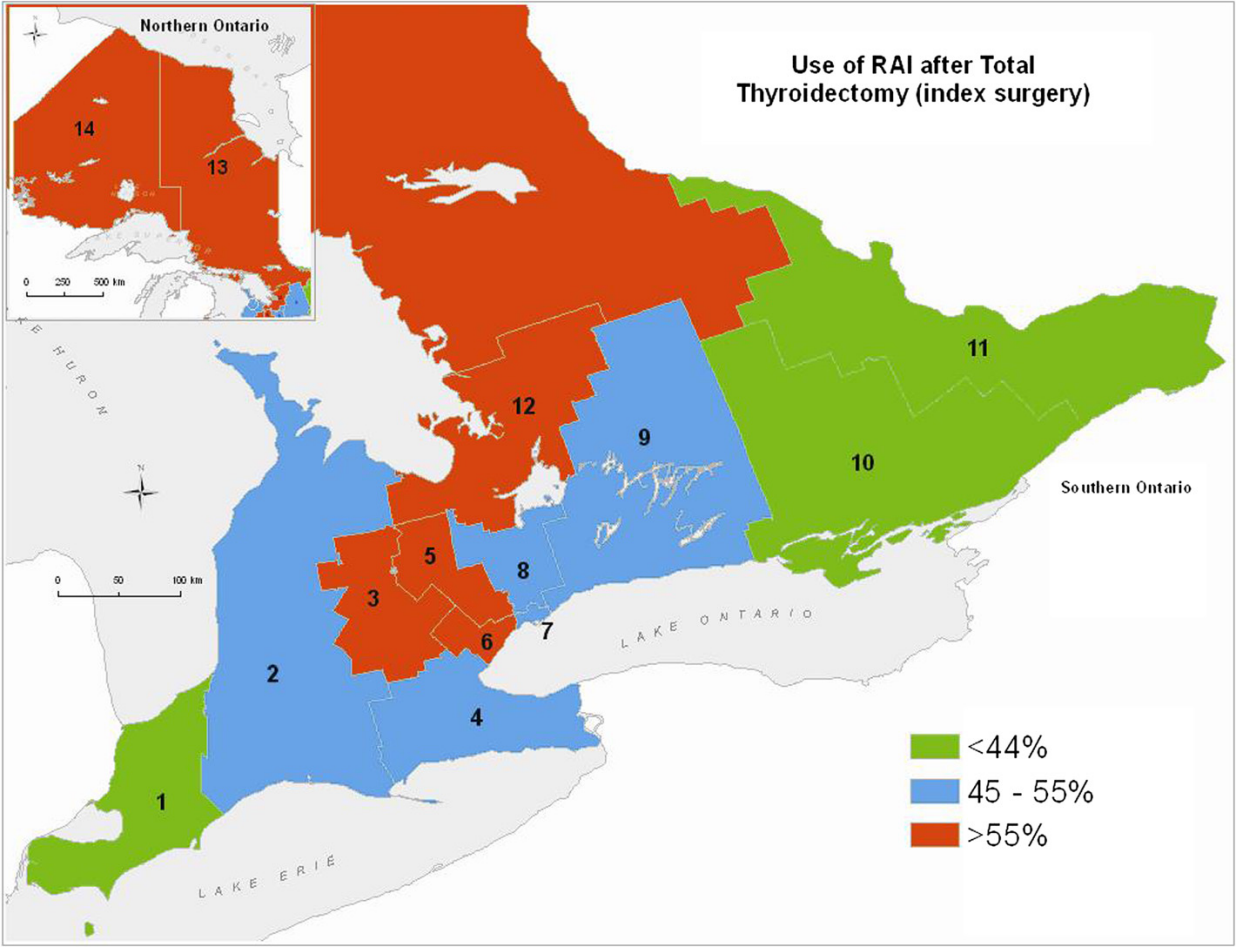
Cases treated with RAI: % of patients who had total thyroidectomy as index surgery by patient LHIN.

The practice of adjuvant RAI, similar to the extent of surgery, varied across the Province. Based on patient address the range of use of post operative RAI varied from 46.1 - 66% (Figure 7) with the highest rates in some of the LHINs with the highest incidence and the highest rates of TT. Due to differences in treatment resources there was considerable patient migration such that the number of cases treated by each LHIN varied from zero (LHINs 3 and 12 had no facility) to 3305 (LHIN 7), with 3 LHINs treating 600-1000 patients and 8 treating 100-400 patients over the 9 years. The large number of cases treated in LHIN 7 corresponds to the 50 RAI specialists who treated the patients, whereas the other LHINs had 1-11 RAI specialists. Higher numbers of RAI physicians in some LHINs reflected more total cases but this was not consistent across the province.

**Figure 8:**
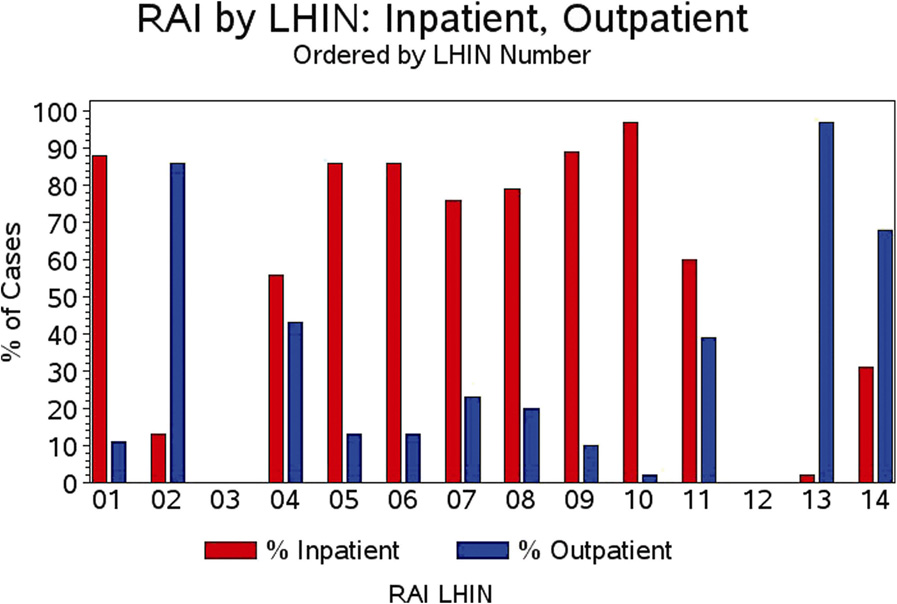
RAI practice by treatment protocol: % In-patient vs % Out-patient RAI based on LHIN of treatment.

RAI can be given as inpatient or as outpatient treatment noting that inpatient RAI may be a surrogate for dose as well in some jurisdictions. Figure 8 presents the RAI regimens by LHIN based on treatment location and regimens also varied by LHIN with 10.3% –91% of patients having in-patient treatment.

Overall 63.4% of the patients having TT as an index surgery and 77.6% of those who had a completion procedure following less-than-total thyroidectomy went on to have RAI.

### Radioactive iodine practitioners

**Figure 9:**
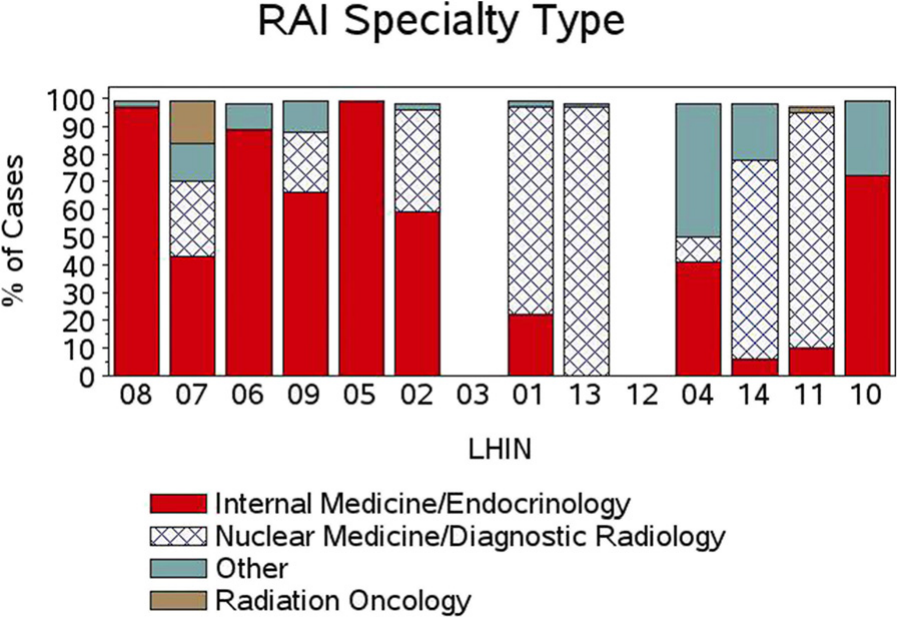
Physician specialty for RAI treatment: % of cases treated by specialty based on treatment LHIN.

Figure 9 demonstrates the variation in specialists treating patients with RAI by LHIN. 53.9% of RAI cases were billed by Internal Medicine Specialists (including Endocrinologists), 27.1% by Radiologists (including Diagnostic and Nuclear Medicine) and 6.97% by Radiation Oncologists (7.6% other and 4.4% missing).

### Overall treatment sequence

Table 1 summarizes the treatment sequence in the first year. 17.2% of patients had LTT only, 27.1% had TT, and 55.5% had TT with RAI. As expected there were considerable differences between LHINs with ranges for LTT (11% - 25%) and TT only (18% - 32%).

**Table 1:** Sequence of treatment over first year for all patients

	**% cases**
**Non-total thyroidectomy alone**	**17.2**
**Non-total thyroidectomy with completion**	**4.3**
**Non-total thyroidectomy with completion and RAI**	**15.9**
**Total thyroidectomy alone**	**22.8**
**Total thyroidectomy with RAI**	**39.6**
	**(100)**
**Non-total thyroidectomy only**	**17.2**
**All total thyroidectomy without RAI**	**27.1**
**All total thyroidectomy with RAI**	**55.5**
	**(100)**

## Discussion

The objective of this project was to create the knowledge foundation on current practice that could be used for informed discussions on the management of thyroid cancer. We found wide variation in all aspects of care including the use of diagnostic tests, initial surgical treatment and RAI treatment along with significant variation in the physician specialties providing care for this group of patients. Overall we found a 4 fold difference in rate of diagnosis by LHIN that correlated with the differences in the use of diagnostic ultrasound of the neck. We confirmed that trans-LHIN patient migration and out-of-region referral patterns were common for both surgery and RAI. We determined that TT was performed more often than LTT in the LHINs of the Greater Toronto region compared to the rest of the province of Ontario and that TT was performed more often by otolaryngologist/head and neck surgeons compared to general surgeons. Similarly RAI was given in different regimens by different specialty physicians across the province. These data confirm that in Ontario there is no agreement on most aspects of the management of TC and as the diagnosis of thyroid cancer is increasing in females, there is an urgent need for policies on extent of surgery and the use of RAI for physicians, patients and the funders of health care systems. Policies however have to be based on evidence not merely consensus, and the further research, based on our identified variations, could generate downstream evidence on the impact of tests and treatments such as further tests, outcomes (relapse, further treatments, further tests, survival) and costs.

The strength of this study is the complete dataset of all patients with TC due to the universal health care in the Province of Ontario, the legislated collection of data from multiple sources on all patients and the availability of centralized administrative datasets.

The limitation of this study is the lack of data on extent of disease. Unlike the SEER dataset in the United States, neither tumor size nor stage for thyroid cancer are available electronically in the Ontario Cancer Registry. Our report is similar to the ICES Surgical Atlas [[6]] – a description of treatments by regions without extent of disease. There is however no reason to suspect that there are wide differences in the proportions of histological types or extent of disease across the LHINs - in particular in the numbers of cases with poorer prognosis cancers. There is no reason to suspect that the vast majority of cases are not small papillary cancers. One potential weakness of the study is the reliability of the extent of surgery data and we used two strategies to reduce error. First we used the hospital procedure codes as our primary data. The CIHI and OHIP datasets have excellent agreement for the coding of common surgical procedures [[7]] but the reliability for TC surgery is not known and the OHIP fee schedule may not reflect some scenarios. Our second strategy to reduce error as well as any potential bias in the codes was to re-examine all patients with non-matching codes and incompatible sequences before deletion.

Practice patterns are often estimated by surveys and the surveys based on case scenarios about practice patterns in thyroid cancer management support our findings on non-agreement and treatment variation. For example, Wu et al. [[8]] reported the results of a survey of 438 thyroid cancer surgeons in the United States on extent of surgery in a low-risk case scenario when the patient had had a hemithyroidectomy. They found 29.7% of surgeons would perform a completion thyroidectomy. Using multiple variable analysis, they found geographic region, case volume and specialty drove more extensive treatment with otolaryngologists more aggressive than general surgeons. Interestingly higher case volume was associated with less aggressive surgery. They concluded that the surgical treatment of papillary micro-carcinoma was inconsistent across different regions and dependent on surgeon’s training. Similarly Sawka et al. [[9]] reported the results of a survey of 486 thyroid cancer specialists in Canada and the United States on the use of RAI in a specific low-risk clinical case scenario. They found no agreement on the indications for treatment across different geographic regions even when the risk level of the case scenario was increased. They reported for the low-risk case scenario that non-university affiliation and physician specialty predicted a stronger recommendation for treatment. Van den Bruel et al. [[10]] reported a survey of thyroidologists in Belgium. The case of a 3 cm FNA suspicious nodule would have been treated with total thyroidectomy in 96% of responders but there was no agreement on extent of neck dissection despite the negative ultrasound. When the nodule was reduced to 0.9 cm low risk case there still was no agreement. Finally Parks et al. [[11]] reported a survey of Canadian otolaryngologists about the management of a case of micropapillary carcinoma. They found 97% of surgeons would do a total thyroidectomy for tumor < 1 cm although there was no agreement on extent of neck dissection. They noted the ATA Guideline for the case was that the patient “may be treated with lobectomy”. These 4 surveys confirm the lack of agreement and identify factors such as geographical regions and specialist type similar to our data.

Our findings are consistent with other population-based research literature on practice patterns in thyroid cancer management. Haymart et al. [[12]] for example, utilized the US National Cancer Database dataset with 189,219 patients with DTC between 1990 and 2008 to study practice patterns for RAI across the US. They found an increasing use of RAI over time for patients after TT. The variables associated with increased use included age (younger), comorbidity (healthier), stage (increasing stage) and increasing hospital case volume. The variables associated with decreasing use were race (African-American), insurance (absence of) and gender (female). They report a complex analysis demonstrating that only small proportions of the variance in practice could be explained by age, gender, race, comorbidity, poverty, insurance, education, rurality, stage, case volume and hospital type. Curiously they found the high unexplained variance extending to include groups such as low-risk younger females with small tumors and older males with larger tumors where indications might have been clearer. They concluded that there was clinical uncertainty on the indications for RAI, that extent of disease did not seem to drive treatment and that overtreatment needed to be considered because of the potential for treatment-induced toxicity and late side effects. Similarly Panigrahi et al. [[13]] used the SEER dataset to examine the national practice pattern for medullary thyroid cancer between 1988 and 2006. They identified 2033 cases and compared their treatments to the ATA guideline published in 2009. They found statistically significant differences in the extent of treatment in different geographic regions of the United States (South, Midwest, Northeast and West) when controlling for tumor size and tumor extent. Both the population based studies found complex relationships with practice.

Variations in the management of other head and neck cancers have been reported within Ontario and compared to Ontario. For example, the treatment of hypopharyngeal cancer for example varied during 1990-2000 across the 8 cancer treatment centers with between-center differences of 85% to 42% having primary radiotherapy and 2% to 43% having primary surgery [[14]]. Similarly large variations in the use of radiotherapy versus primary surgery have been documented between Ontario and the United States in laryngeal cancer [[15],[16]]. Variations in rates of diagnosis and extent of treatment for other cancer sites have been comprehensively described for the Province of Ontario in the ICES Atlas of Cancer Surgery (2008) [[6]]. This Atlas described cancer diagnosis and surgery for 31,457 patients with breast, colon, prostate, lung and gynaecological cancers by LHIN from April 1 2003 to March 31 2004. The objectives of the Atlas were to support regional planning, provide a background for research and improve quality of care. Between-LHIN variations in rates of diagnosis approaching 2-fold were noted in breast, prostate and lung cancer. This study on thyroid cancer has confirmed a 4-fold difference in rate of diagnosis between highest and lowest rate regions. The Atlas proposed that the differences could be related to true differences in case mix related to factors such as smoking rates, to differences in screening or to differences in the use of diagnostic tests. Our data suggests that differences in rates of diagnosis are related to differences in the use of diagnostic tests [[5],[17]] noting that the 4-fold difference between LHINs for thyroid cancer diagnosis compared to the 2-fold differences for other cancer sites could simply be explained by the uncovering of the reservoir of undetected non-progressing or slow growing cancers with increasing access to diagnostic ultrasound [[18]]. The Atlas reported that the age-standardized rates of surgical management were consistent across the province for breast and colon cancer, but not for prostate and lung, and suggested that true differences in population or differences in access might be explanations for the variations in practice. The extent of surgery and the variations in the extent of those surgeries was also reported for some cancers by LHIN such as mastectomy with node resection for breast and for resection with permanent stoma for rectal cancer. Reasons for the variation were postulated to include access to further treatments, patient preference and enthusiasm of the surgeon. Our results for thyroid cancer show considerable patient migration suggesting patient preference or referral bias contribute to differences in extent of both surgery and use of RAI.

Our finding of wide variations in practice are consistent with the ongoing controversies in the management of TC, the existing practice pattern literature based on surveys and the existing practice patterns literature based on populations. There are 4 theories, anchored by clinical research, on treatment rate variations that might help explain these wide differences in opinion. Eddy’s Theory of Uncertainty [[2]] leads from lack of consensus [[1]] where uncertain physicians do not know what is right due to poor evidence, can come to different conclusions about the existing evidence and are often swayed local colleagues who are seen as opinion leaders. The local leaders set standards despite the poor evidence, the uncertain physicians feel safe from criticism and the standards for the region become dogma. Eddy’s theory is about the uncertain physician and the second theory, the theory of Enthusiasm [[19]], is about the “certain” physicians. According to Chasen, physicians who become enthusiasts for a particular treatment, can become blinded to both the uncertainty and the lack of evidence and therefore justify pushing the boundary between Appropriateness and Inappropriateness [[20]]. Appropriateness in the management of TC, might be considered the Guidelines of the American Thyroid Association, but these are consensus statements by experts and clearly Ontario physicians did not support the ATA guidelines of the day for many patients. Chasen’s study and hypothesis was based on high volume surgeons (enthusiasts) for carotid endarterectomy, and as we did find large differences in practice by region as well as specialty, further research is planned to test Chasen’s theory based on the Ontario data on the treatment of TC.

## Conclusion

There is wide variation in all aspects of the management of thyroid cancer in many jurisdictions including Ontario Canada. Differences in treatment produce differences in risk and cost, and differences in rates of diagnosis have the potential to create over diagnosis with over treatment along with the costs of overtreatment. Evidence-based consensus is needed in the management of this disease.

## Competing interests

The authors declare that they have no competing interests.

## Authors’ contributions

SH designed the study, assisted with the data collection, guided the analysis and prepared the manuscript. JI advised on the analysis including clinical aspects and revised the manuscript. PG advised on the analysis and revised the manuscript. DU assisted with data collection and advised on manuscript. All authors read and approved the final manuscript.
